# IMA genome - F14: Draft genome sequences of *Penicillium roqueforti*, *Fusarium sororula, Chrysoporthe puriensis,* and *Chalaropsis populi*

**DOI:** 10.1186/s43008-021-00055-1

**Published:** 2021-03-05

**Authors:** Magriet A. van der Nest, Renato Chávez, Lieschen De Vos, Tuan A. Duong, Carlos Gil-Durán, Maria Alves Ferreira, Frances A. Lane, Gloria Levicán, Quentin C. Santana, Emma T. Steenkamp, Hiroyuki Suzuki, Mario Tello, Jostina R. Rakoma, Inmaculada Vaca, Natalia Valdés, P. Markus Wilken, Michael J. Wingfield, Brenda D. Wingfield

**Affiliations:** 1 Department of Biochemistry, Genetics and Microbiology, Forestry and Agricultural Biotechnology Institute (FABI), University of Pretoria, Private bag X20, Pretoria, 0028 South Africa; 2 Biotechnology Platform, Agricultural Research Council, Onderstepoort, Pretoria, 0110 South Africa; 3 Departamento de Biología, Facultad de Química y Biología, Universidad de Santiago de Chile (USACH), Alameda 3363, Estación Central, 9170022 Santiago, Chile; 4 Department of Plant Pathology, Universidade Federal de Lavras/UFLA, Lavras, MG 37200-000 Brazil; 5 Departamento de Química, Facultad de Ciencias, Universidad de Chile, Las Palmeras 3425, Ñuñoa, Santiago, Chile

**Keywords:** *Fusarium fujikuroi* species complex (FFSC), Colombia, *Pinus tecunumanii*, Eucalyptus leaf pathogen

## Abstract

Draft genomes of *Penicillium roqueforti*, *Fusarium sororula, Chalaropsis populi*, and *Chrysoporthe puriensis* are presented. *Penicillium roqueforti* is a model fungus for genetics, physiological and metabolic studies, as well as for biotechnological applications. *Fusarium sororula* and *Chrysoporthe puriensis* are important tree pathogens, and *Chalaropsis populi* is a soil-borne root-pathogen. The genome sequences presented here thus contribute towards a better understanding of both the pathogenicity and biotechnological potential of these species.

## IMA GENOME – F 14A

### Draft genome sequence of *Penicillium roqueforti* CECT 2905^T^

#### Introduction

*Penicillium roqueforti* is one of the economically most important fungal species within the genus *Penicillium*. This fungus is widely known in the food industry because it is responsible for the ripening of blue cheeses (Chávez et al. 2011). In addition, in recent years, *P. roqueforti* has acquired growing importance as a model fungus for genetics, physiological and metabolic studies, as well as for biotechnological applications (Coton et al. 2020).

**Fig. 1:**
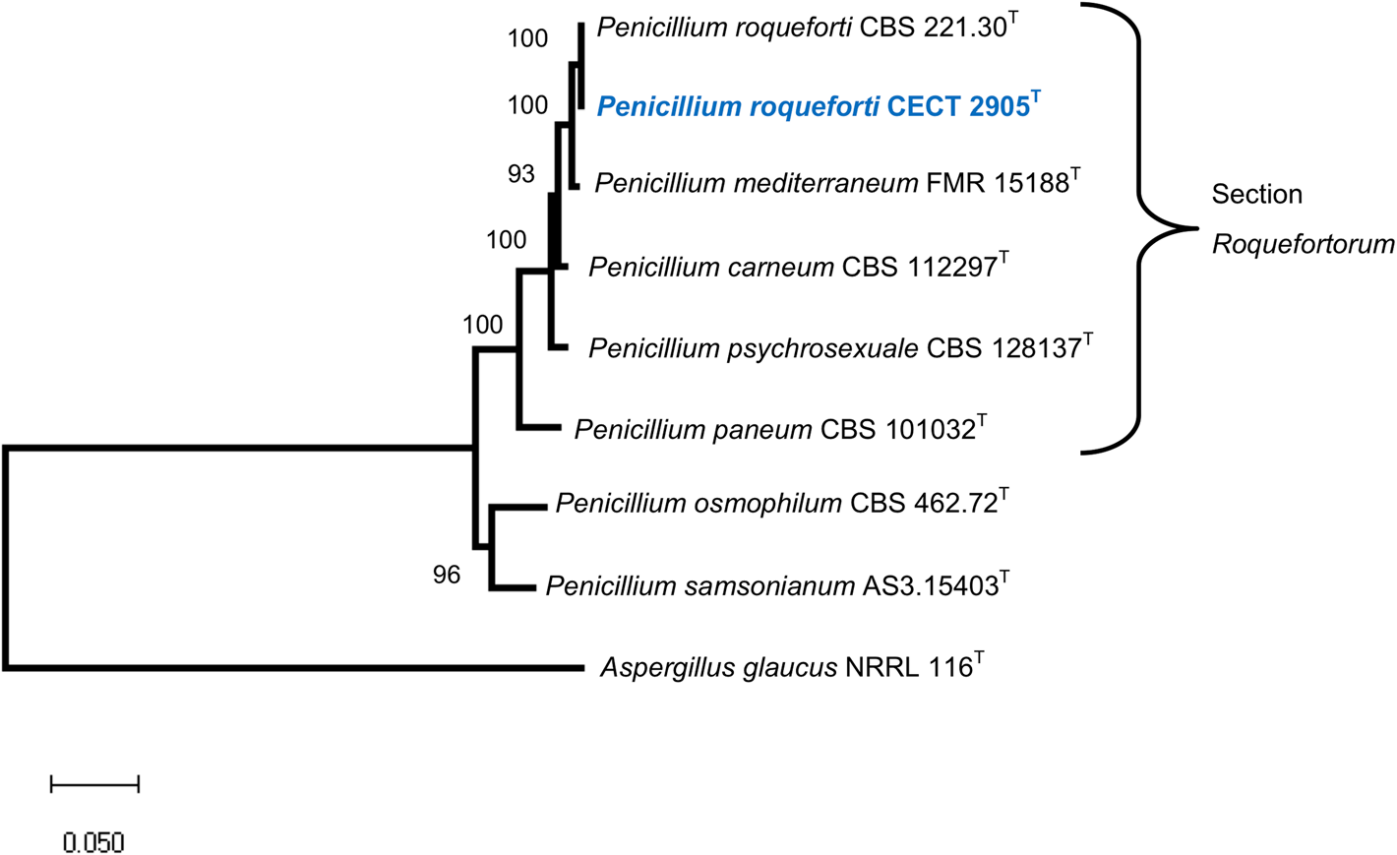
Phylogenetic tree obtained after Maximum Likelihood analysis of the genome sequence of *Penicillium roqueforti* CECT 2905^T^ and related species. The analysis was done as detailed in the Materials and methods section. Bootstrap support values (> 50%) are shown at the nodes (bootstrap iterations = 1000). The tree was rooted using combined *BenA*, *CaM* and *RPB2* regions from *Aspergillus glaucus* NRRL 116^T^. ^T^ = ex-type strain

Phylogenetically, *P. roqueforti* belongs to section *Roquefortorum* within the genus *Penicillium* (Houbraken et al. 2020; Fig. 1). *P. roqueforti* was originally described by Thom (1906), and the nomenclatural type of the species is the neotype IMI 024313 (Frisvad and Samson 2004). From this neotype strain, several ex-type strains have been obtained, which are stored in different culture collections around the world.

Among the ex-type strains obtained from the neotype IMI 024313, *P. roqueforti* CECT 2905^T^ is one of the most widely used. Many molecular studies have been carried out in this ex-type strain, including the analysis of regulatory genes of development and metabolism (García-Rico et al. 2009; Gil-Durán et al. 2015; Torrent et al. 2017; Rojas-Aedo et al. 2018), and importantly, *P. roqueforti* CECT 2905^T^ has been used to demonstrate the functionality of all biosynthetic gene clusters (BGCs) for the production of secondary metabolites characterized so far in this fungal species (Hidalgo et al. 2014; Kosalková et al. 2015; Del-Cid et al. 2016; Fernández-Bodega et al. 2017; Rojas-Aedo et al. 2017).

Owing to the importance of *P. roqueforti* CECT 2905^T^ as model for molecular studies and BGCs characterization, the availability of its genome would be very useful. Consequently, we report this genome resource here. The availability of the genome of *P. roqueforti* CECT 2905^T^ will facilitate future studies on the functional characterization of further BGCs and genes related with the regulation of development and metabolism in this important fungal species.

#### Sequenced strain

**USA**: Connecticut: Storrs, isol. ex French Roquefort cheese, 1904, *C. Thom* (CECT 2905^T^, ex-type strain from the neotype IMI 024313).

#### Nucleotide sequence accession number

The genome sequence of *Penicillium roqueforti* CECT 2905^T^ has been deposited in the DDBJ/ENA/ GenBank databases under the accession number MSQC00000000; Bio project PRJNA351232; Bio-sample SAMN05951162. The version described in this paper is version MSQC00000000.

#### Materials and methods

*Penicillium roqueforti* CECT 2905^T^ was grown on CM broth as described before (Gil-Durán et al. 2015). Mycelium was harvested, washed with NaCl 0.9%, and high-molecular weight DNA was extracted exactly as was described by Bainbridge et al. (1990).

High-molecular weight DNA from *P. roqueforti* CECT 2905^T^ was sequenced using the Illumina HiSeq 2000 platform at Macrogen (Seoul, Korea). A pair-end library with insert sizes of 550 bp was prepared using TruSeq DNA PCR Free kit, and used to generate 101 bp length reads. The quality of the data obtained was assessed using FastQC. Low quality data and adapters were removed with Trimmomatic v. 0.36 (Bolger et al. 2014). Genome assembly of high quality Illumina raw reads was performed with Bowtie2 v. 2.4.1 (Langmead and Salzberg 2012), using the genome of *P. roqueforti* FM164 (Cheeseman et al. 2014) as reference. The final assembly was subjected to completeness assessment using Benchmarking Universal Single-Copy Orthologs program (BUSCO v. 4.0.6; Seppey et al. 2019), utilizing *Eurotiales* odb10 dataset. Genes were predicted with AUGUSTUS v. 3.3.3 (Stanke et al. 2008) using the training dataset from *Aspergillus nidulans*. Finally, in order to identify BGCs, anti-SMASH fungal version v. 5 (Blin et al. 2019) was conducted with default parameters.

The phylogenetic analysis of *P. roqueforti* CECT 2905^T^ and related species was done using combined β-tubulin (*BenA*), calmodulin (*CaM*) and RNA polymerase II second largest subunit (*RPB2*) regions (Guevara-Suarez et al. 2020). Sequences from related species were obtained from GenBank accessions reported by Houbraken et al. (2020), whereas those from *P. roqueforti* CECT 2905^T^ were extracted from the sequence genome. Maximum Likelihood analysis was done in MegaX (Kumar et al. 2018) under GTR + G model.

#### Results and discussion

The phylogenetic tree based on the concatenated *BenA*, *CaM* and *RPB2* regions confirmed that the sequenced genome belongs to the species *P. roqueforti*. *P. roqueforti* CECT 2905^T^ clustered together with *P. roqueforti* CBS 221.30^T^ with a bootstrap support of 100%. In addition, this *P. roqueforti* clade was clearly separated from the other five species within the *Roquefortorum* section (Fig. 1).

Table 1 summarizes the main metrics of the assembled genome sequence of *P. roqueforti* CECT 2905^T^. The assembled draft genome has a total length of 26.1 Mb corresponding to 1168 contigs with an N50 value of 70,366 bp, L50 value of 112, and an average GC content of 48.9%. AUGUSTUS predicted 9015 protein coding genes, with an average gene density of 345.4 genes per 1 Mb. BUSCO analysis reported a completeness score of 98.8% based on the identification of 4141 complete and 18 fragmented genes from a total of 4191 *Eurotiales* genes searched. The estimated genome size of *P. roqueforti* CECT 2905^T^ is comparable to that of other *P. roqueforti* strains found in databases, namely JCM 22842 (27.1 Mb; GenBank accession number BCID00000000), UASWS P1 (27.9 Mb; GenBank accession number JNNS01000000) and FM164 (28 Mb; Cheeseman et al. 2014).

**Table 1:** General characteristics of the genome of *Penicillium roqueforti* CECT 2905^T^

**Assembly metrics**	
Total bases	2,019,762,619
Read count	22,375,066
GC (%)	48.9
Number of contigs	1168
Assembly size (Mb)	26.1
N50 (bp)	70,366
L50	112
Predicted genes models	9015
Gene density (genes per Mb)	345.4
BUSCO completeness	98.8%
**Biosynthetic gene clusters (BGCs)**	
PKS type I	9
PKS type I-NRPS	3
NRPS	7
NRPS-indole	1
NRPS-like fragments	6
NRPS-like fragment-indole	1
Terpene	3
Beta-lactone	2
Indole	1
Siderophore	1

The BGCs prediction performed with anti-SMASH fungal version yielded a total of 34 regions associated with biosynthesis of secondary metabolites. The BGCs found correspond to type I polyketide synthases (PKS), non-ribosomal peptide synthetases (NRPS), NRPS-like fragments, PKS-NRPS, NRPS-indole, NRPS-like-indole, terpene, siderophore, and beta-lactone. The number of BGCs predicted in this work is in good agreement with previous estimation by Coton et al. (2020) who suggested that strains of *P. roqueforti* would contain between 34 and 37 BGCs. To date, only six BGCs have been functionally characterized in *P. roqueforti* CECT 2905^T^ (Hidalgo et al. 2014; Kosalková et al. 2015; Del-Cid et al. 2016; Fernández-Bodega et al. 2017; Rojas-Aedo et al. 2017), so the availability of its draft genome, informed in this paper, will facilitate future studies of functional characterization of further BGCs in this important fungus.

*Authors:* Natalia Valdés, Mario Tello, Inmaculada Vaca, Carlos Gil-Durán, Gloria Levicán, and Renato Chávez*

*Contact: renato.chavez@usach.cl

## IMA GENOME – F 14B

### Draft genome assembly of *Fusarium sororula*

#### Introduction

*Fusarium* species within the *Fusarium fuikuroi* species complex (FFSC) are plant pathogens of various cultivated crops of economic importance (Kvas et al. 2009; Leslie and Summerell 2006). As such, numerous FFSC species have their genomes sequenced, with the first, *F. verticillioides*, published in 2010 (Ma et al. 2010). Currently, 51 FFSC species have genome sequences publicly available (www.ncbi.nlm.nih.gov).

In a study exploring the diversity of FFSC species associated with *Pinus* species in Colombia, five new species were described (Herron et al. 2015). One of these, *Fusarium sororula*, was isolated from diseased *P. tecunumanii* seedlings that displayed symptoms of wilt, shoot dieback and roots with lesions that were resin soaked (Steenkamp et al. 2012). These are all symptoms typical of infection by the pitch canker pathogen, *F. circinatum*. This new species was able to cause disease on susceptible *P. patula*, at similar levels as *F. circinatum* (Herron et al. 2015). *Fusarium sororula* is consequently a threat to global commercial forestry and the availability of its genome sequence will contribute to studies aimed at better understanding its biology and genetics.

#### Sequenced strain

**Colombia:** Angela Maria, Santa Rosa, 75°36^′^21^″^W 4°49^′^18^″^N, isolated from diseased *Pinus tecunumanii* seedlings*,* 2006, *C.A. Rodas* (CMW 25513; FCC 5425; PREM63211-dried culture) (Herron et al. 2015).

#### Nucleotide sequence accession number

This Whole Genome Shotgun project has been deposited at DDBJ/ENA/GenBank under the accession JACWFA000000000. The version described in this paper is version JACWFA010000000.

#### Materials and methods

The *Fusarium sororula* isolate was grown on half strength potato dextrose agar (BD Difco™) and genomic DNA was extracted according to the protocol of Möller et al. 1992. One pair-end library (550 bp insert size, read length of 250 bp) was generated using the Illumina HiSeq 2500 platform at Macrogen (Seoul, Korea). Poor quality and duplicate reads were removed using Qiagen Genomics Workbench v 20.0.4 (CLCBio, Aarhus). Reads were assembled using SPAdes v 3.13.0 (Bankevich et al. 2012). Completeness of the genome assembly was evaluated with BUSCO v 4.0.6 (Seppey et al. 2019), using the hypocreales dataset. Annotation was done with the MAKER annotation pipeline (Cantarel et al. 2008) using Augustus (Stanke et al. 2006a, 2006b), Genemark ES (Ter-Hovhannisyan et al. 2008) and SNAP (Korf 2004). Gene model data from *F. circinatum* (Wingfield et al. 2012), *F. fujikuroi* (Wiemann et al. 2013), *F. verticillioides* and *F. graminearum* (Ma et al. 2010), as well as *F. mangiferae* and *F. proliferatum* (Niehaus et al. 2017), were included as additional evidence.

#### Results and discussion

**Fig. 2:**
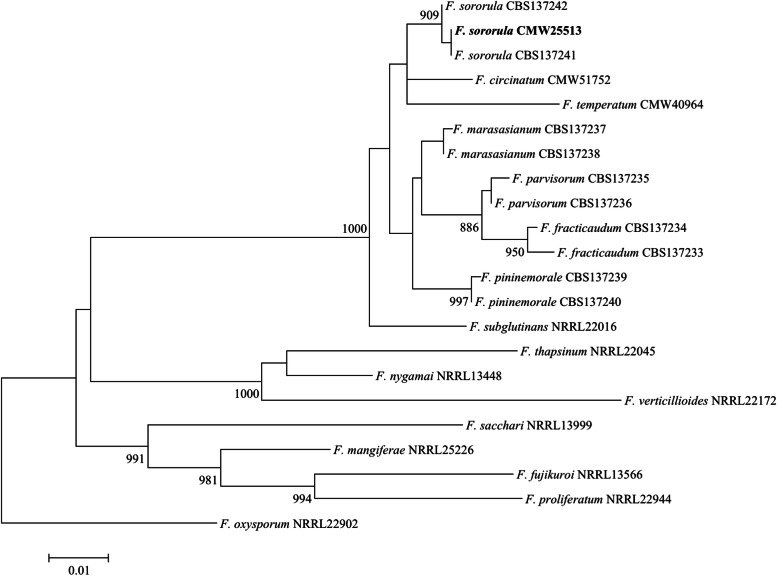
Maximum Likelihood tree based on the partial gene sequences of translation elongation factor 1-α and β-tubulin (Herron et al. 2015). Sequence alignments were assembled with MAFFT v 7.472 (Katoh et al. 2019). The program jModelTest v 2.1.10 (Darribo et al. 2012) was used to determine the best-fit substitution model (GTR + I + G substitution model) with gamma correction (Tavare 1986). A maximum Likelihood (ML) phylogenetic analysis was performed using PhyML v 3.1 (Guindon et al. 2010). Values at branch nodes are the bootstrapping confidence values with those ≥85% shown. The *F. sororula* isolate sequenced in this study is indicated in bold

The genome sequence of *F. sororula* was assembled into 328 scaffolds with a total genome size of 47,806,863 bp. The N50 value was 1,089,458 bp and the genome had a G + C content of 45.99%. BUSCO analyses showed that the assembly was 99.9% complete [4486 complete and single-copy BUSCOs, 7 complete and duplicated BUSCOs; 2 fragmented BUSCOs, 6 missing BUSCOs; *n* = 4494]. A total of 15,668 open reading frames (orfs) were predicted using the MAKER annotation pipeline, with a gene density of 327.74 orfs/Mb. Sequence comparisons indicated that all twelve known chromosomes typical for species in the FFSC are present. Phylogenetic analysis of sequences from the sequenced genome confirmed the taxonomic identity of the sequenced *Fusarium* strain as *F. sororula* (Fig. 2).

This genome announcement is the third for a *Fusarium* species isolated from *Pinus* species in Colombia. The two previously sequenced and assembled genomes where for *F. fracticaudum* (Wingfield et al. 2018a) and *F. pininemorale* (Wingfield et al. 2017). This genome assembly for *F. sorula* is comparable to other American clade species from the FFSC isolated from *Pinus* species (Table 2; Wingfield et al. 2018a; Wingfield et al. 2017; Wingfield et al. 2018b), with genome size, G + C content and gene density all in a similar range.

**Table 2:** Genome statistics for *Fusarium sororula* and its close relatives

	Genome size (Mb)	GC content (%)	Predicted orfs^**a**^	Gene density (orfs/Mb)	References
*F. sororula*	47.81	45.99	15,688	327.74	This study
*F. fracticaudum*	45.80	47.56	14,136	308.67	Wingfield et al. (2018a)
*F. pininemorale*	47.78	45.98	15,455	323.47	Wingfield et al. (2017)
*F. circinatum*	45.10	46.97	15,091	334.61	Wingfield et al. (2018b)

^a^Determined as described in text

*Authors:* Lieschen De Vos^*^, Magriet A. van der Nest, **Quentin C. Santana, Emma T. Steenkamp, Brenda D. Wingfield**

^*^Contact: lieschen.bahlmann@fabi.up.ac.za

## IMA GENOME – F 14C

### Draft nuclear genome assembly of *Chalaropsis populi,* the second genome from the genus *Chalaropsis*

#### Introduction

*Chalaropsis populi* is a soil-borne root-pathogen in the family *Ceratocystidaceae* (Paulin-Mahady et al. 2002; de Beer et al. 2014). The first record of *Ch. populi* is from the early 1970’s where it was isolated from the bark of *Populus* and *Salix* spp. in Belgium (Veldeman 1971). This species was referred to as *Chalaropsis populi*, but no validly published description ever appeared (Kiffer and Delon 1983). Kiffer and Delon (1983) subsequently validated the name *Chalaropsis populi,* as *Chalara populi,* but in 2002 this species was once again redescribed and included in *Thielaviopsis* as *T. populi* (Paulin-Mahady et al. 2002). Most recently, the genus *Chalaropsis* was re-established and now includes three named species: *Ch. ovoidea, Ch. populi,* and *Ch. thielavioides*, although some evidence supports the recognition of a fourth taxon (de Beer et al. 2014).

Species of *Chalaropsis* are not considered of significant ecological or economic importance despite being predominantly isolated from diseased plant material (de Beer et al. 2014). The type species of the genus, *Ch. thielavioides,* is commonly associated with post-harvest moulding of carrot (Weber and Tribe 2004; Milosavljević et al. 2015; Xu et al. 2020), although the economic impact of the disease is negligible. *Chalaropsis ovoidea* is predominantly isolated from *Fagus* trees (and discoloured planks produced from the wood), but can occasionally be found in *Quercus* species as well (Nag Raj and Kendrick 1976; Kraj and Kowalski 2005). *Chalaropsis populi* was originally isolated from brown spots associated with trunk scab disease in *Populus* and *Salix*, prompting its description as a cambium killer (Kiffer and Delon 1983). A subsequent study also found *Ch. populi* in combination with other fungi from diseased roots of *Populus* and *Euramericana* trees, but the authors considered it a weak pathogen (Szabó and Harrington 2004).

The low level of importance of these pathogens has resulted in very little research effort focussed on *Chalaropsis* species. Nevertheless, the taxonomic positioning of *Chalaropsis* as a sister genus to many important pathogens in the genera *Berkeleyomyces, Ceratocystis,* and *Endoconidiophora* make this group of interest. A draft genome for *Ch. thielavioides* was generated by the RIKEN Center for Life Science Technologies, Division of Genomic Technologies, and is publicly available (GCA_001599435.1) (JCM-Riken 2016). In the current study, a draft genome sequence for *Ch. populi* is presented to accompany that of *Ch. thielavioides*. It is hoped that the availability of two genome sequences for *Chalaropsis* species will support future studies on comparative genomics, while also addressing the taxonomic complexities associated with asexual fungi.

**Fig. 3:**
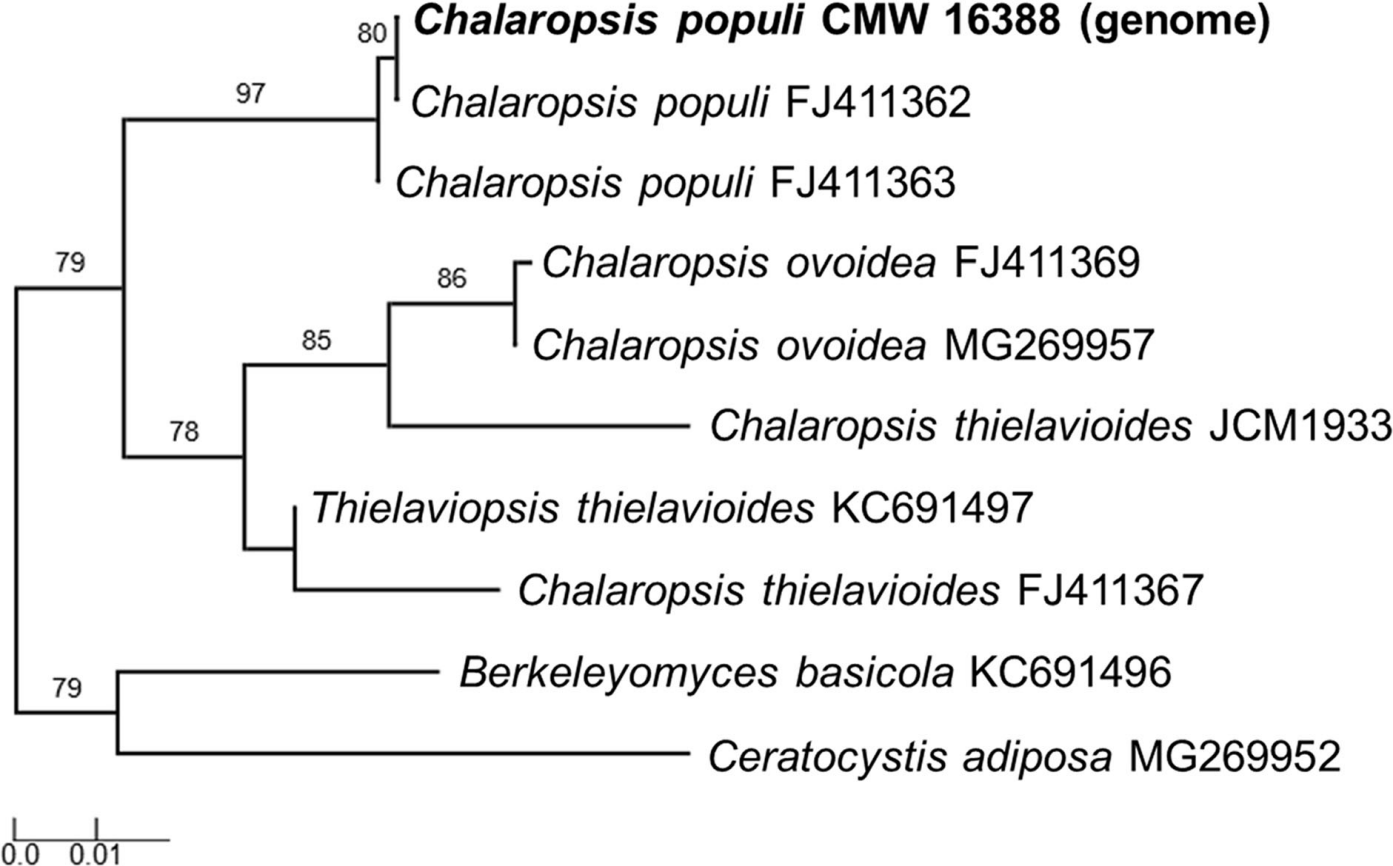
A Maximum Likelihood phylogeny based on the beta-tubulin gene from species of *Chalaropsis*. This analysis confirms the identity of the genome assembly presented here (shown in bold) as *Chalaropsis populi*. Interestingly, the publicly available *Ch. thielavioides* JCM 1933 genome (Genbank accession: GCA_001599435) grouped in the same clade as *Ch. ovoidea*. *Ceratocystis adiposa* and *Berkeleyomyces basicola* were used as outgroups, and the results for the approximate likelihood ratio test for branch support are shown as percentages

#### Sequenced strain

**Belgium**: Gent: Moerzeke Populetum, isol. from necrosis in *Populus gelrica,* 1970, *R. Veldeman* (CMW 26388, CBS 486.71, CBS H-10141 - dried culture).

#### Nucleotide sequence accession number

This Whole Genome Shotgun project for *Chalaropsis populi* isolate CMW 26388 has been deposited at DDBJ/ENA/GenBank under the accession JADILG000000000. The version described in this paper is version JADILG010000000.

#### Materials and methods

*Chalaropsis populi* isolate CMW 26388 was obtained from the culture collection (CMW) of the Forestry and Agricultural Biotechnology Institute (FABI) based at the University of Pretoria and grown on 2% malt extract agar (MEA: 2% w/v, Biolab, South Africa) at 25 °C for the duration of the study. Genomic DNA was isolated from a 14-day old culture grown on a cellophane sheet using the DNeasy Plant Mini Kit (Qiagen, Germany). The isolated DNA was sent to the Agricultural Research Council Biotechnology Platform (ARC-BTP; Pretoria, South Africa) where it was used to prepare a pair-end library with an insert size of 500 bp. An Illumina HiSeq 2500 (Illumina, San Diego, CA) was used to generate 125 bp length reads from both ends of the insert.

The raw reads generated were imported and trimmed (using the Trim Sequences command and default values) in CLC Genomics Workbench v. 20.0.3 (CLCBio, Aarhus) before being used in a de novo assembly to generate a draft genome sequence. The untrimmed paired reads were also used for read-error correction and assembly with SPAdes v. 3.14.0 (Bankevich et al. 2012) using custom K-values (21, 33, 55, 77), applying the “careful” option to reduce mismatches and including the CLC-generated scaffolds as untrusted contigs. An estimation of the number of protein coding genes in the *Ch. populi* genome was made using the AUGUSTUS de novo prediction software with *Fusarium graminearum* gene models (Stanke et al. 2006a, 2006b; Keller et al. 2011). General genome statistics (genome length, GC content, N50 and L50 values) for the *Ch. populi* assembly were calculated using QUAST v. 5.0.1 (Gurevich et al. 2013), while both this genome and the *Ch. thielavioides* JCM 1933 assembly (JCM-Riken 2016) was assessed for completeness using the Benchmarking Universal Single Copy Orthologs tool (BUSCO v. 4.0.6) (Simão et al. 2015) using both the Fungi_odb10 and Ascomycota_odb10 datasets.

The publicly available genome sequence for *Ch. thielavioides* JCM 1933 was retrieved from the genome repository at the National Center for Biotechnology Information (NCBI) (JCM-Riken 2016). The beta-tubulin gene was extracted from the draft genome assemblies of both *Ch. populi* CMW 26388 and *Ch. thielavioides* JCM 1933 using CLC Genomics Workbench. These were used together with published sequences from *Ch. ovoidea*, *Ch. thielavioides, Ch. populi*, *Ceratocystis adiposa* and *Berkeleyomyces basicola* in a phylogenetic analysis to confirm the identity of the sequenced strains (Fig. 3). To do this, the one click mode phylogeny online tool (Dereeper et al. 2008, 2010) that included MUSCLE alignment (Edgar 2004), curation via Gblocks (Castresana 2000), PhyML steps (Guindon and Gascuel 2003) and a Maximum Likelihood test for branch support was used (Anisimova and Gascuel 2006). The tree was rooted using *Ceratocystis adiposa* and *Berkeleyomyces basicola.*

#### Results and discussion

The draft genome sequence of *Ch. populi* had a length of 23,877,278 bp present in 2158 contigs, of which 1398 were larger than 1000 bp. The genome had a GC content of 52.56%, an average coverage of 81x, a N50 value of 29,267 bp and a L50 value of 239. AUGUSTUS predicted 6654 protein coding genes, while BUSCO analysis reported a completeness score of 96.7 and 98.0% for *Ch. populi* for the respective *Ascomycota* and *Fungi* BUSCO datasets. This was based on the analysis of 1706 and 758 orthologs for the *Ascomycota* and *Fungi* datasets respectively, where 1650 and 743 were present and complete, while 48 and 13 copies were completely absent. The comparative BUSCO analysis for the *Ch. thielavioides* JCM 1933 genome assembly indicated a 84.1% (1435 complete and 266 missing BUSCOs) and 85.8% (650 complete and 108 missing BUSCOs) completeness for the *Ascomycota* and *Fungi* dataset.

Phylogenetic analysis using the beta-tubulin gene from the sequenced genome confirmed the identity of the isolate as *Ch. populi* (Fig. 3), although the publicly available genome sequence for *Chalaropsis thielavioides* JCM 1933 grouped closer to *Ch. ovoidea* than known *Ch. thielavioides* strains. When compared to the *Ch. thielavioides* genome, *Ch. populi* has a similar genome size (23,8 Mb for *Ch. populi* vs 23,3 Mb for *Ch. thielavioides*), although it is more fragmented (2158 vs 252 contigs) (JCM-Riken 2016). This is supported by the N50 values (29,267 bp for *Ch. populi* vs 161,617 bp for *Ch. thielavioides*). However, the *Ch. populi* genome was more complete based on the BUSCO assessments.

The *Ch. populi* draft assembly is the second genome sequence available for a *Chalaropsis* sp. This will support research efforts aimed at understanding the biology of these understudied fungal pathogens (Weber and Tribe 2004). For example, all three known species of *Chalaropsis* are considered asexual (de Beer et al. 2014), a stark contrast to the predominantly sexual species in the *Ceratocystidaceae*. Much work has been focussed on sexual reproduction in the family (e.g. Wilken et al. 2017; Nel et al. 2018; Simpson et al. 2018), and the availability of two genome sequences for putatively asexual members will be a valuable addition to this ongoing project. Together with the *Ch. populi* sequence, there are now 30 species residing in *Ceratocystidaceae* of which the genomes have been sequenced and these include representatives of ten genera (https://www.ncbi.nlm.nih.gov/datasets/genomes/?txid=1028423&term=Ceratocystidaceae&utm_source=assembly&utm_medium=referral&utm_campaign=:assemb). These sequences provide the opportunity to perform family-level analyses seeking to answer questions regarding speciation processes, host adaptations, and comparative genomics. They will also provide a basis for further functional studies (e.g. Sayari et al. 2019; Wilson et al. 2020) in *Ceratocystidaceae.*

*Authors:* Jostina R. Rakoma, Frances A. Lane, P. Markus Wilken*

* *Contact*: Markus.Wilken@fabi.up.ac.za

## IMA GENOME– F 14D

### Draft genome sequence of *Chrysoporthe puriensis*: the cause of a canket disease on *Eucalyptus* and *Tibouchina*

#### Introduction

The genus *Chrysoporthe* accommodates numerous economically important pathogens of plantation *Eucalyptus* species and other members of *Myrtales* (Gryzenhout et al. 2009). These fungi cause serious stem canker diseases, predominantly in tropical and subtropical parts of the world (Wingfield 2003). *Chrysoporthe puriensis* was first reported causing a stem canker disease on *Tibouchina* spp. in Brazil (Oliveira et al. 2021). Pathogenicity tests showed that *C*. *puriensis* is pathogenic on the hybrid *Eucalyptus grandis* × *E*. *urophylla*, suggesting that the fungus could threaten commercially-grown *Eucalyptus* plantations in South America (Oliveira et al. 2021). Similar to other *Chrysoporthe* spp. causing stem cankers on trees, *C. puriensis* is a potential threat to trees in *Myrtales* grown as non-natives for commercial purposes or where they are native. Countries such as Australia that has a mega-diverse *Myrtales* flora are especially vulnerable to these relatively wide host range pathogens (Burgess and Wingfield 2017); as has been seen for the globally spreading myrtle rust pathogen *Austropuccinia psidii* (Glen et al. 2007; Roux et al. 2016).

Genome sequences are available for three species of *Chrysoporthe*, which infect *Eucalyptus*. These include, *C*. *austroafricana*, *C. cubensis*, and *C*. *deuterocubensis* (Wingfield et al. 2015a, 2015b). The aim of this study was to sequence and assemble the genome of *C*. *puriensis* that will enable comparative genome studies focussed on further understanding the biology of *Chrysoporthe* species and to improve disease management strategies for them.

#### Sequenced strain

**Brazil**: *Minas Gerais*: Silveirânia, *Tibouchina granulosa*, 2018, *M.E.S. Oliveira* (CMW 54409).

#### Sequence accession numbers

The genome sequence of *Chrysoporthe puriensis* (isolate number CMW 54409) has been deposited in DDBJ/EMBL/GenBank databases under the accession numbers CP064894 - CP064907.

#### Materials and methods

Genomic DNA was extracted from freeze-dried mycelium of isolate CMW 54409 grown in malt yeast broth (2% malt extract, 0.5% yeast extract; Biolab, Midrand, South Africa) using the Qiagen® Genomic-tip DNA extraction protocol for plants and fungi. To verify the identification of the isolate, sequencing of the internal transcribed spacer (ITS) region and the partial β-tubulin gene (*tub*1 and *tub*2) was performed. The reference sequences were obtained from GenBank. *Amphilogia gyrosa* was used as an outgroup. Sequence datasets were aligned using an online version of MAFFT v.7 (Katoh and Standley 2013). A maximum likelihood analysis was performed using RAxML (Stamatakis 2014) using the GTR + G substitution model and branch support was calculated using 1000 bootstrap replicates.

Nanopore sequencing was conducted using the MinION sequencing device. The sequencing library was prepared using the Genomic DNA by Ligation (SQK-LSK109) protocol. The library was loaded on a MinION flow cell (R10.3) and sequencing run was carried out for 48 h. Base calling was conducted using ONT Guppy basecalling software v 4.0.14.

Nanopore reads were trimmed using Porechop (v0.2.1, https://github.com/rrwick/Porechop). The genome was assembled using Flye v 2.7 (Kolmogorov et al. 2019). The assembly was polished using Rebaler v0.2.0 (Wick et al. 2019), which runs multiple rounds of Racon v1.4.13 (Vaser et al. 2017) and followed by two rounds of polishing iterations with Medaka v1.0.3 (https://github.com/nanoporetech/medaka). Protein coding gene models were annotated using AUGUSTUS v.3.3 with *Magnaporthe grisea* as the model organism (Stanke and Morgenstern 2005). The assembled genome completeness was evaluated using the Benchmarking Universal Single-Copy Orthologs tool, BUSCO v. 4.1.3 by using the fungal lineage dataset (Simão et al. 2015).

#### Results and discussion

**Fig. 4:**
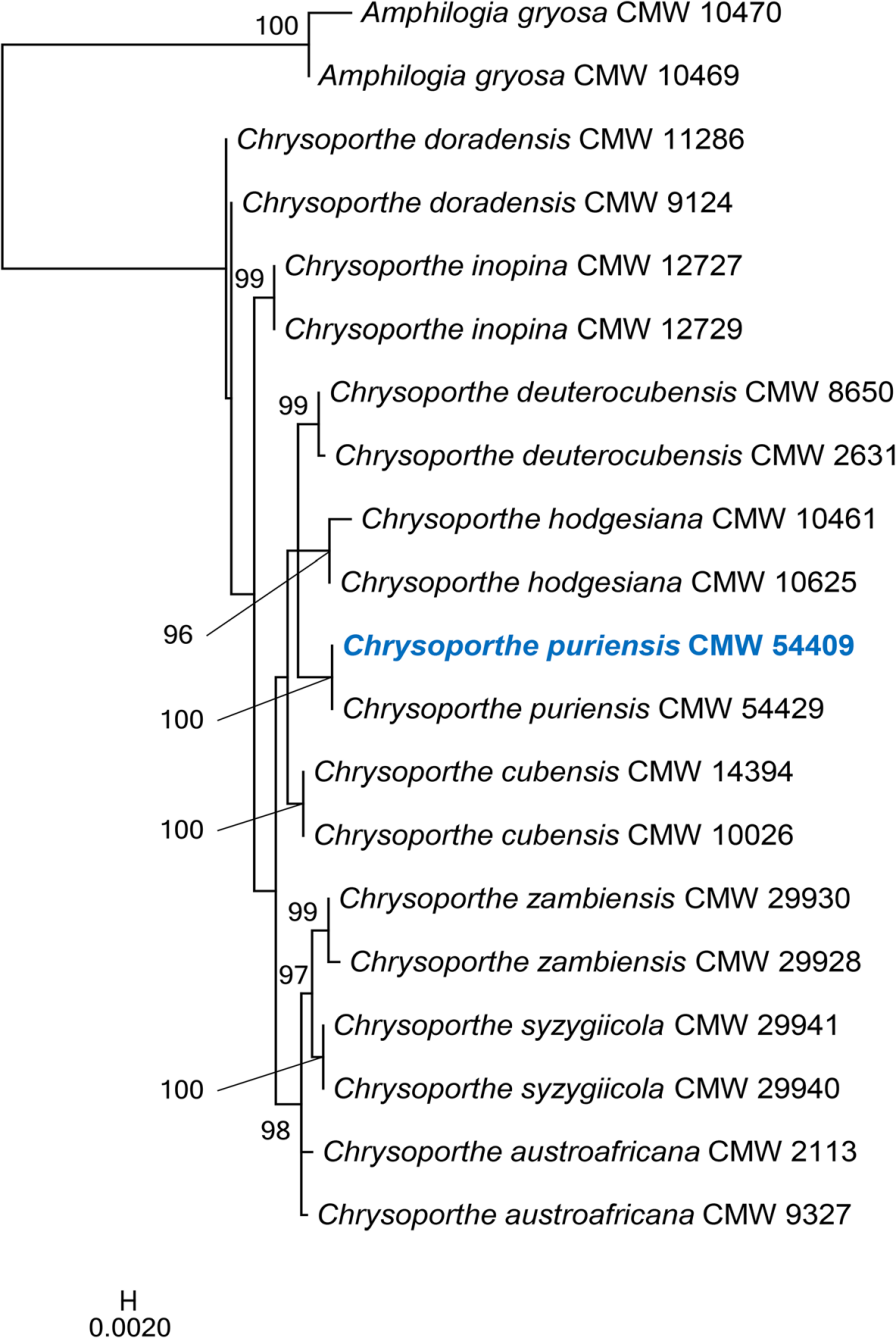
Maximum Likelihood tree based on ITS region and partial gene sequences of *but*1 and *but*2. Bootstrap values ≥65% are shown. The isolates used in this study are indicated in blue and bold

Phylogenetic analysis using three gene regions (ITS, *tub*1, and *tub*2) confirmed the taxonomic identity of isolate CMW 54409 as *C*. *puriensis* (Fig. 4). The assembly of *C*. *puriensis* consisted of 14 contigs, with the N50 of 4.78 Mb and L50 of 5. The calculated genome size was approximately 44.66 Mb and with a CG content of 53.91%. AUGUSTUS predicted 13,166 protein coding gene models in the assembled genome. BUSCO analysis using the sordariomycetes_odb10 dataset indicated the assembled genome to have a 98.3% completeness score. Of the 3817 BUSCO groups searches, 13 BUSCO orthologs were reported to be fragmented and 54 BUSCO groups were reported to be missing.

The estimated genome size and gene number for *C*. *puriensis* is similar to that of other *Chrysoporthe* species: *Chrysoporthe ausroafricana* (44.6 Mb, 13,484) (Wingfield et al. 2015a), *C. cubensis* (42.6 Mb, 13,121) (Wingfield et al. 2015b), *C*. *deuterocubensis* (43.9 Mb; 13,772) (Wingfield et al. 2015b). The draft genome sequence of *C*. *puriensis* generated here will be used for comparative genomics studies as well as to better understand its, biology and role as a tree pathogen.. Furthermore, the genome sequence will be useful for to develop molecular markers for population studies of the species and to determine its origin and pathways of movement in forests.

*Authors:* H. Suzuki, T.A. Duong*, M.A. Ferreira, M.J. Wingfield, B.D. Wingfield

*Contact: Tuan.Duong@fabi.up.ac.za
